# Supplementary material for: The environmental impact of health care for musculoskeletal conditions: A scoping review
Source: PLoS One. 2022 Nov 28;17(11):e0276685. doi: 10.1371/journal.pone.0276685 (PMC9704655; doi:10.1371/journal.pone.0276685)
Supplement: S2 Data — (DOCX) [file pone.0276685.s002.docx]

**S2: Search strategies**

**Database: Ovid MEDLINE(R) and Epub Ahead of Print, In-Process, In-Data-Review & Other Non-Indexed Citations, Daily and Versions(R) <1946 to 2 May 2022 at 9.56pm AEDT>**

--------------------------------------------------------------------------------

1 exp Musculoskeletal Diseases/ (1166679)

2 musculoskeletal.mp. [mp=title, abstract, original title, name of substance word, subject heading word, floating sub-heading word, keyword heading word, organism supplementary concept word, protocol supplementary concept word, rare disease supplementary concept word, unique identifier, synonyms] (78192)

3 rheumatoid.mp. [mp=title, abstract, original title, name of substance word, subject heading word, floating sub-heading word, keyword heading word, organism supplementary concept word, protocol supplementary concept word, rare disease supplementary concept word, unique identifier, synonyms] (154329)

4 osteoarthriti*.mp. [mp=title, abstract, original title, name of substance word, subject heading word, floating sub-heading word, keyword heading word, organism supplementary concept word, protocol supplementary concept word, rare disease supplementary concept word, unique identifier, synonyms] (103758)

5 osteoporo*.mp. [mp=title, abstract, original title, name of substance word, subject heading word, floating sub-heading word, keyword heading word, organism supplementary concept word, protocol supplementary concept word, rare disease supplementary concept word, unique identifier, synonyms] (102765)

6 exp Scleroderma, Systemic/ (22242)

7 scleroderm*.mp. [mp=title, abstract, original title, name of substance word, subject heading word, floating sub-heading word, keyword heading word, organism supplementary concept word, protocol supplementary concept word, rare disease supplementary concept word, unique identifier, synonyms] (29557)

8 exp Vasculitis/ (101242)

9 vasculiti*.mp. [mp=title, abstract, original title, name of substance word, subject heading word, floating sub-heading word, keyword heading word, organism supplementary concept word, protocol supplementary concept word, rare disease supplementary concept word, unique identifier, synonyms] (49829)

10 gout.mp. [mp=title, abstract, original title, name of substance word, subject heading word, floating sub-heading word, keyword heading word, organism supplementary concept word, protocol supplementary concept word, rare disease supplementary concept word, unique identifier, synonyms] (18820)

11 spondylarthriti*.mp. [mp=title, abstract, original title, name of substance word, subject heading word, floating sub-heading word, keyword heading word, organism supplementary concept word, protocol supplementary concept word, rare disease supplementary concept word, unique identifier, synonyms] (4067)

12 lupus.mp. [mp=title, abstract, original title, name of substance word, subject heading word, floating sub-heading word, keyword heading word, organism supplementary concept word, protocol supplementary concept word, rare disease supplementary concept word, unique identifier, synonyms] (96753)

13 exp Lupus Erythematosus, Systemic/ (64346)

14 exp Low Back Pain/ (24921)

15 (back pain or neck pain or shoulder pain or elbow pain or heel pain or foot pain or ankle pain or wrist pain or knee pain or hip pain or forearm pain, or arm pain, or hand pain, or thigh pain).mp. [mp=title, abstract, original title, name of substance word, subject heading word, floating sub-heading word, keyword heading word, organism supplementary concept word, protocol supplementary concept word, rare disease supplementary concept word, unique identifier, synonyms] (112332)

16 exp Arthroplasty, Replacement/ (65752)

17 arthroplast*.mp. [mp=title, abstract, original title, name of substance word, subject heading word, floating sub-heading word, keyword heading word, organism supplementary concept word, protocol supplementary concept word, rare disease supplementary concept word, unique identifier, synonyms] (101634)

18 joint replacement.mp. [mp=title, abstract, original title, name of substance word, subject heading word, floating sub-heading word, keyword heading word, organism supplementary concept word, protocol supplementary concept word, rare disease supplementary concept word, unique identifier, synonyms] (8154)

19 hip replacement.mp. [mp=title, abstract, original title, name of substance word, subject heading word, floating sub-heading word, keyword heading word, organism supplementary concept word, protocol supplementary concept word, rare disease supplementary concept word, unique identifier, synonyms] (12320)

20 knee replacement.mp. [mp=title, abstract, original title, name of substance word, subject heading word, floating sub-heading word, keyword heading word, organism supplementary concept word, protocol supplementary concept word, rare disease supplementary concept word, unique identifier, synonyms] (9990)

21 shoulder replacement.mp. [mp=title, abstract, original title, name of substance word, subject heading word, floating sub-heading word, keyword heading word, organism supplementary concept word, protocol supplementary concept word, rare disease supplementary concept word, unique identifier, synonyms] (729)

22 exp Arthroscopy/ (26879)

23 Orthopedics/ (22882)

24 Physical Therapists/ (2678)

25 physical therap*.mp. [mp=title, abstract, original title, name of substance word, subject heading word, floating sub-heading word, keyword heading word, organism supplementary concept word, protocol supplementary concept word, rare disease supplementary concept word, unique identifier, synonyms] (61298)

26 physiotherap*.mp. [mp=title, abstract, original title, name of substance word, subject heading word, floating sub-heading word, keyword heading word, organism supplementary concept word, protocol supplementary concept word, rare disease supplementary concept word, unique identifier, synonyms] (31270)

27 Rheumatologists/ (544)

28 rheumatolog*.mp. [mp=title, abstract, original title, name of substance word, subject heading word, floating sub-heading word, keyword heading word, organism supplementary concept word, protocol supplementary concept word, rare disease supplementary concept word, unique identifier, synonyms] (37815)

29 Chiropractic/ (3377)

30 chiropract*.mp. [mp=title, abstract, original title, name of substance word, subject heading word, floating sub-heading word, keyword heading word, organism supplementary concept word, protocol supplementary concept word, rare disease supplementary concept word, unique identifier, synonyms] (7685)

31 Osteopathic Physicians/ (378)

32 osteopath*.mp. [mp=title, abstract, original title, name of substance word, subject heading word, floating sub-heading word, keyword heading word, organism supplementary concept word, protocol supplementary concept word, rare disease supplementary concept word, unique identifier, synonyms] (8173)

33 Sports Medicine/ (11308)

34 Podiatry/ (2363)

35 podiatr*.mp. [mp=title, abstract, original title, name of substance word, subject heading word, floating sub-heading word, keyword heading word, organism supplementary concept word, protocol supplementary concept word, rare disease supplementary concept word, unique identifier, synonyms] (4374)

36 Occupational Therapists/ (538)

37 occupational therap*.mp. [mp=title, abstract, original title, name of substance word, subject heading word, floating sub-heading word, keyword heading word, organism supplementary concept word, protocol supplementary concept word, rare disease supplementary concept word, unique identifier, synonyms] (22172)

38 rehabilitation physician*.mp. [mp=title, abstract, original title, name of substance word, subject heading word, floating sub-heading word, keyword heading word, organism supplementary concept word, protocol supplementary concept word, rare disease supplementary concept word, unique identifier, synonyms] (312)

39 Physiatrists/ (95)

40 physiatrist*.mp. [mp=title, abstract, original title, name of substance word, subject heading word, floating sub-heading word, keyword heading word, organism supplementary concept word, protocol supplementary concept word, rare disease supplementary concept word, unique identifier, synonyms] (1358)

41 (Physical Medicine and Rehabilitation).mp. [mp=title, abstract, original title, name of substance word, subject heading word, floating sub-heading word, keyword heading word, organism supplementary concept word, protocol supplementary concept word, rare disease supplementary concept word, unique identifier, synonyms] (3676)

42 or/1-41 (1753326)

43 environmental health.mp. or exp Environmental Health/ (34362)

44 environmental monitoring.mp. or exp Environmental Monitoring/ (160263)

45 environmental impact.mp. (8756)

46 ecological sustainability.mp. (238)

47 exp "Conservation of Natural Resources"/ (107106)

48 exp Environmental Pollution/ or exp Climate Change/ (621027)

49 life cycle assessment.mp. (3319)

50 carbon footprint.mp. or exp Carbon Footprint/ (1836)

51 ecological footprint.mp. (516)

52 exp Medical Waste/ (3626)

53 exp Recycling/ (5866)

54 or/43-53 (705304)

55 42 and 54 (7868)

56 exp animals/ not humans.sh. (5001776)

57 55 not 56 (7145)

**Database: Embase Classic+Embase <1947 to 2 May 2022 at 10.09pm AEDT >**

--------------------------------------------------------------------------------

1 exp musculoskeletal disease/ (2746848)

2 musculoskeletal.mp. [mp=title, abstract, heading word, drug trade name, original title, device manufacturer, drug manufacturer, device trade name, keyword heading word, floating subheading word, candidate term word] (180653)

3 rheumatoid.mp. [mp=title, abstract, heading word, drug trade name, original title, device manufacturer, drug manufacturer, device trade name, keyword heading word, floating subheading word, candidate term word] (281266)

4 osteoarthriti*.mp. [mp=title, abstract, heading word, drug trade name, original title, device manufacturer, drug manufacturer, device trade name, keyword heading word, floating subheading word, candidate term word] (173088)

5 osteoporo*.mp. [mp=title, abstract, heading word, drug trade name, original title, device manufacturer, drug manufacturer, device trade name, keyword heading word, floating subheading word, candidate term word] (185116)

6 exp systemic sclerosis/ (36463)

7 scleroderm*.mp. [mp=title, abstract, heading word, drug trade name, original title, device manufacturer, drug manufacturer, device trade name, keyword heading word, floating subheading word, candidate term word] (35854)

8 exp vasculitis/ (146549)

9 vasculiti*.mp. [mp=title, abstract, heading word, drug trade name, original title, device manufacturer, drug manufacturer, device trade name, keyword heading word, floating subheading word, candidate term word] (90376)

10 gout.mp. [mp=title, abstract, heading word, drug trade name, original title, device manufacturer, drug manufacturer, device trade name, keyword heading word, floating subheading word, candidate term word] (31607)

11 spondylarthriti*.mp. [mp=title, abstract, heading word, drug trade name, original title, device manufacturer, drug manufacturer, device trade name, keyword heading word, floating subheading word, candidate term word] (11744)

12 lupus.mp. [mp=title, abstract, heading word, drug trade name, original title, device manufacturer, drug manufacturer, device trade name, keyword heading word, floating subheading word, candidate term word] (166303)

13 exp systemic lupus erythematosus/ (109987)

14 exp low back pain/ (67615)

15 (back pain or neck pain or shoulder pain or elbow pain or heel pain or foot pain or ankle pain or wrist pain or knee pain or hip pain or forearm pain, or arm pain, or hand pain, or thigh pain).mp. [mp=title, abstract, heading word, drug trade name, original title, device manufacturer, drug manufacturer, device trade name, keyword heading word, floating subheading word, candidate term word] (204301)

16 exp replacement arthroplasty/ (37008)

17 arthroplast*.mp. [mp=title, abstract, heading word, drug trade name, original title, device manufacturer, drug manufacturer, device trade name, keyword heading word, floating subheading word, candidate term word] (120889)

18 joint replacement.mp. [mp=title, abstract, heading word, drug trade name, original title, device manufacturer, drug manufacturer, device trade name, keyword heading word, floating subheading word, candidate term word] (11905)

19 hip replacement.mp. [mp=title, abstract, heading word, drug trade name, original title, device manufacturer, drug manufacturer, device trade name, keyword heading word, floating subheading word, candidate term word] (26509)

20 knee replacement.mp. [mp=title, abstract, heading word, drug trade name, original title, device manufacturer, drug manufacturer, device trade name, keyword heading word, floating subheading word, candidate term word] (31130)

21 shoulder replacement.mp. [mp=title, abstract, heading word, drug trade name, original title, device manufacturer, drug manufacturer, device trade name, keyword heading word, floating subheading word, candidate term word] (1317)

22 exp arthroscopy/ (36131)

23 orthopedics/ (26842)

24 physiotherapist/ (25561)

25 physical therap*.mp. [mp=title, abstract, heading word, drug trade name, original title, device manufacturer, drug manufacturer, device trade name, keyword heading word, floating subheading word, candidate term word] (45583)

26 physiotherap*.mp. [mp=title, abstract, heading word, drug trade name, original title, device manufacturer, drug manufacturer, device trade name, keyword heading word, floating subheading word, candidate term word] (140564)

27 rheumatologist/ (7433)

28 rheumatolog*.mp. [mp=title, abstract, heading word, drug trade name, original title, device manufacturer, drug manufacturer, device trade name, keyword heading word, floating subheading word, candidate term word] (127401)

29 chiropractic/ (5003)

30 chiropract*.mp. [mp=title, abstract, heading word, drug trade name, original title, device manufacturer, drug manufacturer, device trade name, keyword heading word, floating subheading word, candidate term word] (8908)

31 osteopathic physician/ (456)

32 osteopath*.mp. [mp=title, abstract, heading word, drug trade name, original title, device manufacturer, drug manufacturer, device trade name, keyword heading word, floating subheading word, candidate term word] (10941)

33 sports medicine/ (20180)

34 podiatry/ (2944)

35 podiatr*.mp. [mp=title, abstract, heading word, drug trade name, original title, device manufacturer, drug manufacturer, device trade name, keyword heading word, floating subheading word, candidate term word] (5619)

36 occupational therapist/ (8203)

37 occupational therap*.mp. [mp=title, abstract, heading word, drug trade name, original title, device manufacturer, drug manufacturer, device trade name, keyword heading word, floating subheading word, candidate term word] (38725)

38 rehabilitation physician*.mp. [mp=title, abstract, heading word, drug trade name, original title, device manufacturer, drug manufacturer, device trade name, keyword heading word, floating subheading word, candidate term word] (549)

39 physiatrist/ (821)

40 physiatrist*.mp. [mp=title, abstract, heading word, drug trade name, original title, device manufacturer, drug manufacturer, device trade name, keyword heading word, floating subheading word, candidate term word] (2524)

41 (Physical Medicine and Rehabilitation).mp. [mp=title, abstract, heading word, drug trade name, original title, device manufacturer, drug manufacturer, device trade name, keyword heading word, floating subheading word, candidate term word] (12967)

42 or/1-41 (3375938)

43 environmental health.mp. or exp environmental health/ (48450)

44 environmental monitoring.mp. or exp environmental monitoring/ (97535)

45 environmental impact.mp. or exp environmental impact/ (108381)

46 exp environmental sustainability/ or ecological sustainability.mp. (6431)

47 exp environmental protection/ (96217)

48 exp pollution/ (430918)

49 exp climate change/ (48686)

50 exp life cycle assessment/ (6269)

51 carbon footprint.mp. or exp carbon footprint/ (10433)

52 Medical waste.mp. or exp hospital waste/ (4002)

53 exp recycling/ (30948)

54 or/43-53 (710243)

55 42 and 54 (6128)

56 (rat or rats or mouse or mice or swine or porcine or murine or sheep or lambs or pigs or piglets or rabbit or rabbits or cat or cats or dog or dogs or cattle or bovine or monkey or monkeys or trout or marmoset$1).ti. and animal experiment/ (1148207)

57 Animal experiment/ not (human experiment/ or human/) (2413280)

58 or/56-57 (2472330)

59 55 not 58 (5898)

**Google Scholar and Google searches**

**Searches were performed between 2 May and 12 May 2022**

| **Keywords used for search** | **Number of relevant records found using Google** | **Number of relevant records found using Google Scholar** |
| --- | --- | --- |
| life cycle assessment hand | 0 records | 0 records |
| life cycle assessment wrist | 0 records | 0 records |
| life cycle assessment elbow | 0 records | 0 records |
| life cycle assessment shoulder | 0 records | 0 records |
| life cycle assessment foot | 0 records | 0 records |
| life cycle assessment ankle | 0 records | 0 records |
| life cycle assessment knee | 1 record [101] | 4 records [94, 95, 101, 105] |
| life cycle assessment hip | 0 records | 0 records |
| life cycle assessment spine | 1 record [103] | 1 record [103] |
| life cycle assessment spinal | 1 record [101] | 1 record [103] |
| life cycle assessment hand surgery | 3 records [83, 85, 103] | 11 records [61, 83-85, 90, 103, 122, 127-129, 131] |
| life cycle assessment wrist surgery | 4 records [83-85, 103] | 2 records [83, 128] |
| life cycle assessment elbow surgery | 0 records | 1 record [90] |
| life cycle assessment shoulder surgery | 0 records | 1 record [90] |
| life cycle assessment foot surgery | 1 record [103] | 0 records |
| life cycle assessment ankle surgery | 0 records | 1 record [129] |
| life cycle assessment knee surgery | 2 records [101, 103] | 4 records [70, 94, 95, 101] |
| life cycle assessment hip surgery | 1 record [103] | 0 records |
| life cycle assessment spine surgery | 1 record [103] | 1 record [103] |
| life cycle assessment spinal surgery | 1 record [103] | 2 records [103, 129] |
| life cycle assessment surgical | 2 records [90, 103] | 2 records [84, 90] |
| life cycle assessment surgical implant | 3 records [103, 125, 129] | 2 records [96, 103] |
| life cycle assessment orthopaedic surgery | 3 records [85, 103, 125] | 6 records [58, 84, 94, 128, 129, 131] |
| life cycle assessment orthopedic surgery | 2 records [103, 125] | 12 records [67, 70, 84, 85, 90, 94, 101, 103, 104, 128, 129, 131] |
| life cycle assessment joint arthroplasty | 1 record [101] | 6 records [70, 74, 83, 94, 95, 129] |
| life cycle assessment joint arthroscopy | 1 record [103] | 0 records |
| life cycle assessment joint replacement | 1 record [101] | 0 records |
| life cycle assessment telehealth | 1 record [97] | 0 records |
| life cycle assessment telemedicine | 1 record [97] | 1 record [97] |
| sustainability hand surgery | 4 records [120, 121, 125, 128] | 3 records [90, 127, 129] |
| sustainability wrist surgery | 2 records [127, 128] | 1 record [128] |
| sustainability elbow surgery | 0 records | 0 records |
| sustainability shoulder surgery | 0 records | 1 record [90] |
| sustainability foot surgery | 1 record [108] | 2 records [42, 129] |
| sustainability ankle surgery | 1 record [108] | 1 record [129] |
| sustainability knee surgery | 1 record [125] | 1 record [129] |
| sustainability hip surgery | 3 records [31, 49, 92] | 2 records [42, 129] |
| sustainability spine surgery | 1 record [103] | 0 records |
| sustainability spinal surgery | 1 record [103] | 1 record [129] |
| sustainability surgical | 1 record [50] | 0 records |
| sustainability surgical implant | 4 records [35, 96, 103, 129] | 2 records [103, 129] |
| sustainability orthopaedic surgery | 5 records [31, 42, 96, 125, 129] | 3 records [42, 126, 129] |
| sustainability orthopedic surgery | 6 records [42, 96, 104, 125, 126, 129] | 2 records [90, 129] |
| sustainability joint arthroplasty | 6 records [31, 49, 64, 101, 117, 125] | 1 records [82] |
| sustainability joint arthroscopy | 3 records [79, 92, 125] | 0 records |
| sustainability joint replacement | 1 record [125] | 0 records |
| sustainability telehealth | 2 records [54, 75] | 0 records |
| sustainability telemedicine | 2 records [75, 76] | 0 records |
| environmental sustainability hand surgery | 6 records [50, 84, 121, 125, 128, 129] | 6 records [90, 103, 122, 127-129] |
| environmental sustainability wrist surgery | 4 records [50, 121, 125, 128] | 1 record [128] |
| environmental sustainability elbow surgery | 0 records | 1 record [90] |
| environmental sustainability shoulder surgery | 0 records | 3 records [89, 90, 121] |
| environmental sustainability foot surgery | 2 records [49, 108] | 1 record [129] |
| environmental sustainability ankle surgery | 5 records [49, 50, 53, 108, 129] | 2 records [83, 108, 129] |
| environmental sustainability knee surgery | 1 record [125] | 8 records [80, 89, 94, 95, 101, 121, 129, 131] |
| environmental sustainability hip surgery | 8 records [80, 89, 94, 95, 101, 121, 129, 131] | 1 record [67, 129] |
| environmental sustainability spine surgery | 0 records | 1 record [103] |
| environmental sustainability spinal surgery | 1 record [129] | 3 records [78, 91, 129] |
| environmental sustainability surgical | 2 records [89, 129] | 1 record [92] |
| environmental sustainability surgical implant | 3 records [96, 103, 125] | 3 records [35, 103, 129] |
| environmental sustainability orthopaedic surgery | 6 records [67, 84, 121, 125, 128, 129] | 1 record [129] |
| environmental sustainability orthopedic surgery | 5 records [67, 96, 121, 125, 129] | 7 records [82-84, 90, 129-131] |
| environmental sustainability joint arthroplasty | 3 records [49, 121, 125] | 2 records [94, 95] |
| environmental sustainability joint arthroscopy | 2 records [107, 129] | 3 records [80, 82, 129] |
| environmental sustainability joint replacement | 2 records [121, 125] | 1 record [67] |
| environmental sustainability telehealth | 1 record [76] | 0 records |
| environmental sustainability telemedicine | 1 record [76] | 3 records [75-77] |
| environmental impact hand | 0 records | 1 record [85] |
| environmental impact wrist | 0 records | 0 records |
| environmental impact elbow | 0 records | 0 records |
| environmental impact shoulder | 0 records | 0 records |
| environmental impact foot | 2 records [107, 108] | 1 record [62] |
| environmental impact ankle | 3 records [62, 107, 108] | 1 record [62] |
| environmental impact knee | 4 records [27, 95, 101, 125] | 4 records [94, 95, 101, 105] |
| environmental impact hip | 3 records [79, 92, 125] | 2 records [79, 92] |
| environmental impact spine | 3 records [86, 101, 103] | 0 records |
| environmental impact spinal | 0 records | 3 records [78, 86, 103] |
| carbon footprint hand | 1 record [131] | 1 record [131] |
| carbon footprint wrist | 1 record [120] | 1 record [128] |
| carbon footprint elbow | 0 records | 0 records |
| carbon footprint shoulder | 1 record [66] | 2 records [55, 66] |
| carbon footprint foot | 0 records | 0 records |
| carbon footprint ankle | 2 records [107, 108] | 5 records [96, 107, 108, 118, 129] |
| carbon footprint knee | 1 record [101] | 10 records [55, 70, 74, 86, 100, 101, 113, 124, 129, 131] |
| carbon footprint hip | 3 records [49, 92, 121] | 3 records [86, 113, 124] |
| carbon footprint spine | 2 records [86, 101] | 5 records [70, 74, 86, 101, 103] |
| carbon footprint spinal | 2 records [86, 101] | 6 records [70, 74, 78, 86, 101, 103] |
| carbon footprint hand surgery | 8 records [58, 61, 85, 97, 120-122, 125] | 12 records [84, 85, 90, 97, 100, 120-122, 127-129, 131] |
| carbon footprint wrist surgery | 8 records [61, 85, 97, 101, 120, 121, 125, 131] | 2 records [120, 128] |
| carbon footprint elbow surgery | 1 record [120] | 1 record [90] |
| carbon footprint shoulder surgery | 3 records [55, 66, 121] | 5 records [55, 66, 89, 90, 121] |
| carbon footprint foot surgery | 3 records [53, 101, 108] | 4 records [50, 85, 108, 129] |
| carbon footprint ankle surgery | 1 record [108] | 5 records [83, 93, 96, 108, 129] |
| carbon footprint knee surgery | 4 records [55, 101, 121, 125] | 15 records [49, 55, 70, 74, 86, 89, 93, 94, 100, 101, 113, 120, 121, 129, 131] |
| carbon footprint hip surgery | 4 records [92, 120, 121, 125] | 11 records [49, 86, 93, 98, 100, 113, 120, 121, 124, 127, 129] |
| carbon footprint spine surgery | 3 records [78, 86, 101] | 5 records [70, 74, 86, 103, 129] |
| carbon footprint spinal surgery | 2 records [86, 101] | 7 records [70, 74, 78, 86, 101, 103, 129] |
| carbon footprint surgical | 4 records [50, 101, 125, 131] | 3 records [97, 101, 131] |
| carbon footprint surgical implant | 4 records [49, 96, 103, 125] | 8 records [66, 83, 86, 96, 100, 103, 129, 131] |
| carbon footprint orthopaedic surgery | 7 records [58, 96, 101, 120, 121, 125, 129] | 9 records [58, 66, 96, 100, 113, 120, 122, 129, 131] |
| carbon footprint orthopedic surgery | 6 records [58, 101, 120, 121, 125, 129] | 14 records [70, 78, 84-86, 90, 93, 101, 120, 122, 127-129, 131] |
| carbon footprint joint arthroplasty | 4 records [101, 120, 121, 125] | 11 records [49, 70, 74, 93-95, 101, 121, 124, 129, 131] |
| carbon footprint joint arthroscopy | 1 record [101] | 2 records [128, 129] |
| carbon footprint joint replacement | 3 records [101, 121, 125] | 5 records [70, 74, 100, 101, 124] |
| carbon footprint telehealth | 5 records [53, 57, 75, 77, 97] | 3 records [75, 77, 97] |
| carbon footprint telemedicine |  |  |
|  | 4 records [53, 75, 77, 97] | 6 records [54, 75-77, 97, 131] |

**Total number of records considered for inclusion from Google and Google Scholar keyword searches:** 59 records [27, 31, 35, 42, 49, 50, 53-55, 57, 58, 61, 64, 66, 67, 70, 74-80, 82-86, 89-98, 100, 101, 103-105, 107, 108, 113, 117, 118, 120-122, 124-131]

**Number of duplicate records with MEDLINE and Embase search results:** 13 records [27, 80, 85, 89, 92, 94, 96, 98, 101, 107, 117, 118, 124]

**Number of excluded full-text publications found using Google and Google Scholar keyword searches (see Table S1 for reasons):** 9 records [31, 35, 42, 49, 50, 53-55, 57]

**Number of records from Google and Google Scholar keyword searches that were classified as awaiting assessment (see Table S2 for reasons):** 5 records [58, 61, 62, 64, 66]

**Number of included publications from Google and Google Scholar keyword searches:**

11 studies (19 reports of studies) [67, 70, 74-79, 83, 84, 86, 90, 91, 95, 97, 100, 103-105]

8 editorials (9 reports of editorials) [82, 108, 113, 120-122, 125-127]

4 literature reviews [128-131]

**Total:** 23 publications (32 reports of publications)
