# Supplementary material for: The environmental impact of health care for musculoskeletal conditions: A scoping review
Source: PLoS One. 2022 Nov 28;17(11):e0276685. doi: 10.1371/journal.pone.0276685 (PMC9704655; doi:10.1371/journal.pone.0276685)
Supplement: S1 Table — (DOCX) [file pone.0276685.s003.docx]

**S1 Table. Excluded studies**

| **Study** | **Reasons for exclusion** |
| --- | --- |
| **Aldoori *et al.* 2021 [50]** | Wrong setting. An editorial that encourages environmentally sustainable practice for surgery but does not explicitly discuss any musculoskeletal condition or type of orthopaedic surgery. |
| **Aoyama 2014 [25]** | Wrong topic. Described the link between human activity and climate change. Provided suggestions for occupational therapists to promote environmental sustainability. |
| **Baca 2009 [26]** | Wrong topic. This publication discussed a role for occupational therapists to implement refurbished technology devices for programs aimed to improve the functional capabilities of disabled, geriatric and underserved members of the community. |
| **Banerjee & Maric 2021 [27]** | Wrong topic. Discussed the negative impacts of oral NSAIDs used for musculoskeletal pain on human, aquatic and ecosystems health, and the role of physiotherapy as a sustainable alternative to NSAIDs. |
| **Campion *et al.* 2015 [51]** | Wrong setting. This life cycle assessment did not report carbon footprint data specific to musculoskeletal conditions. |
| **Dennis *et al.* 2015 [28]** | Wrong topic. This article linked environmental sustainability issues with occupational therapy philosophy. Discussed that by using a sustainability lens with professional reasoning, occupational therapists can integrate sustainability into their practice. |
| **Epstein 2005 [29]** | Wrong topic. This editorial paper discussed microorganisms and their role in the human body. |
| **Foo 2016 [30]** | Wrong topic. A letter to the editor that highlighted the impact of fluctuating weather conditions on human morbidity and mortality, and suggested that physiotherapists can play a role by raising awareness of climate change impacts. |
| **Hall & Dunstan 2022 [31]** | Wrong topic. This review article describes ‘day case’ total hip replacements as a solution to meet the large demands for lower limb orthopaedic surgeries. |
| **Hofmann & Stössel 1996 [32]** | Wrong topic. A review that discussed the biological, musculoskeletal and psychosocial hazards of health care. |
| **Howe 1974 [33]** | Wrong topic. Discussed how various external factors such as weather conditions, food supply and atmospheric pollution can impact patient health. |
| **Hrachovec 1969 [34]** | Wrong topic. Not related to the environmental impact of health care or health support services for musculoskeletal conditions. |
| **Kumar 2021 [35]** | Wrong topic. This scoping review explores regulations for medical devices and their environmental impact but does not report musculoskeletal medical devices specifically. |
| **Lieb 2020 [36]** | Wrong topic. Not related to the environmental impact of health care or health support services for musculoskeletal conditions. |
| **Lieb 2022 [37]** | Wrong topic. Not related to the environmental impact of health care or health support services for musculoskeletal conditions. |
| **Maric & Nicholls 2021 [38]** | Wrong topic. Provides an outline of environmental physiotherapy based on planetary health, environmental ethics and multispecies justice. |
| **Masino *et al.* 2010 [54]** | Unclear population. No specific data for musculoskeletal conditions were presented. |
| **McAlister *et al.* 2022 [55]** | Unclear population. No specific carbon footprint data were reported for imaging that is commonly requested for musculoskeletal conditions (e.g. x-ray of lumbar spine, ultrasound of the rotator cuff, MRI of the knee etc.). |
| **McGain *et al.* 2015 [56]** | Unclear population. No specific data for musculoskeletal conditions were presented. Unclear how many musculoskeletal surgeries were performed for this waste audit. |
| **Mower & Thompson 2020 [39]** | Wrong topic. Reviews the costs of wasted medicine dispensed to people with lower leg fracture but does not consider the environmental impact. |
| **Nicolet 2022 [57]** | Unclear population. No carbon footprint data were presented for musculoskeletal conditions specifically. |
| **Potteiger *et al.* 2017a [40]** | Wrong topic. Examined athletic trainers’ attitudes toward and perceptions of environmentally sustainable practices. |
| **Potteiger *et al.* 2017b [41]** | Wrong topic. Examined the knowledge of athletic trainers on green techniques such as conservation through recycling, purchasing products and adequate disposal of medication. |
| **Pradhan *et al.* 2021 [42]** | Wrong topic. Explored whether virtual phone consultations were a feasible option for patients and orthopaedic clinicians compared to face-to-face consultations. |
| **Prasad *et al.* 2021 [53]** | Wrong population. In-patients were not being treated for musculoskeletal conditions specifically. |
| **Raymond *et al.* 2020 [52]** | Wrong setting. Not related to the environmental impact of health care or health support services for musculoskeletal conditions. |
| **Riley 1989 [43]** | Wrong topic. This study measured the amount of radiation exposure received by a surgeon and first assistant during from fluoroscopic procedures. |
| **Sanders *et al.* 1993 [44]** | Wrong topic. This study measured the amount of radiation exposure received by an orthopaedic surgeon’s hands during fluoroscopic procedures. |
| **Skubik-Peplaski 2016 [45]** | Wrong topic. This observational study investigated how an occupational therapist’s physical environment influenced the interventions they provided to patients for stroke rehabilitation therapy. |
| **Soroceanu *et al.* 2011 [46]** | Wrong topic. A waste audit that reported the costs associated with spine surgery. The authors did not measure the environmental impact of health care or health support services for spine surgery. |
| **Taylor *et al.* 2022 [47]** | Wrong topic. Predominantly focusses on the climate-related health effects impacting people with disabilities and how this affects the physiatrist profession. |
| **Wagman 2014 [48]** | Wrong topic. Explored the relationship between occupations and climate change. |
| **Wyssusek *et al.* 2019 [49]** | Wrong topic. A narrative literature review that investigated how current practices in the operating room affect the environment and how this can be improved in future. No specific data relating to the environmental impact of health care or health support services for musculoskeletal conditions were reported. |
