# Supplementary material for: The environmental impact of health care for musculoskeletal conditions: A scoping review
Source: PLoS One. 2022 Nov 28;17(11):e0276685. doi: 10.1371/journal.pone.0276685 (PMC9704655; doi:10.1371/journal.pone.0276685)
Supplement: S2 Table — (DOCX) [file pone.0276685.s004.docx]

**S2 Table. Studies awaiting assessment**

| **Study** | **Reason for classification** |
| --- | --- |
| **Abihssira *et al.* [58]** | Data from this conference poster presentation may be incomplete and have not been published in a peer-reviewed journal. |
| **Fort *et al.* 2022 [59]** | Results data from this conference abstract may be incomplete and have not been published in a peer-reviewed journal. |
| **Gillies & Arnaud 2021 [60]** | Data from this conference poster presentation may be incomplete and have not been published in a peer-reviewed journal. |
| **Masmejean & Abihssira 2022 [61]** | Data from this conference poster presentation may be incomplete and have not been published in a peer-reviewed journal. |
| **Mirkouei *et al.* 2017**  **[62]** | This conference paper includes methods, results, discussion and conclusion sections; however, it has not been published in a peer-reviewed journal and may be incomplete. |
| **Parkinson *et al.* 2021 [63]** | Data from this conference poster presentation may be incomplete and have not been published in a peer-reviewed journal. |
| **Pavlou 2010 [64]** | This conference abstract on the environmental impact of large joint arthroplasty contained incomplete data. |
| **Reilly 2021 [65]** | This conference abstract contained insufficient data on the environmental impact of oral paracetamol versus intravenous pain relief prescribed to children presenting for orthopaedic surgery. |
| **Rougereau *et al.* 2022 [66]** | Data from this abstract may be incomplete. |
