# Supplementary material for: The environmental impact of health care for musculoskeletal conditions: A scoping review
Source: PLoS One. 2022 Nov 28;17(11):e0276685. doi: 10.1371/journal.pone.0276685 (PMC9704655; doi:10.1371/journal.pone.0276685)
Supplement: S3 Table — (DOCX) [file pone.0276685.s005.docx]

**S3 Table. Characteristics and findings of editorials**

| **Author (year)** | **Country of author/s** | **Topic** | **Focus** | **Conclusions** |
| --- | --- | --- | --- | --- |
| **Banerjee 2020 [113]** | India | Physiotherapy (carbon footprint) | This grey literature article discussed some evidence on greenhouse gas emissions related to hospital energy consumption. Highlights how physiotherapy may be used pre-operatively to improve patient recovery from surgery to reduce the environmental impact associated with post-operative hospital stays. | Concludes that there is a lack of research to determine whether physiotherapy interventions can reduce the environmental impact of post-operative hospital stays, but highlights the potential based on several studies. |
| **Borg 2021 [120]** | United Kingdom | Hand surgery (environmental impact) | Discusses pre-, intra- and post-operative interventions that can be implemented for hand surgery to minimise material waste, thereby reducing costs and carbon emissions associated with hand surgery. | Outlines that despite possible logistical challenges, hospital administrators should try and implement changes to current hand surgery practice to reduce material waste. These changes will result in financial and environmental benefits. |
| **Bruyneel 2020 [119]** | Switzerland | Physiotherapy (environmental impact) | Contends that it is time to reconsider the relationship between health care (physiotherapy in particular) and the environment. | Suggests actions to limit the environmental impact of physiotherapy based on 4 principles:   - Development of prevention - Promotion of the autonomy of individuals’ health care - Improving treatment effectiveness - Using low-polluting treatments such as physiotherapy may reduce the need for treatments with a higher environmental impact (e.g. surgery, drugs) |
| **Chan *et al.* 2021 [121]** | United Kingdom | Orthopaedic surgery | A grey literature article that highlights the threat of climate change as a planetary and public health issue. Provides background information relating to the environmental impact of surgical operations, supported by peer-reviewed examples. | Concludes that surgical practice should adopt sustainable models of care based on available evidence. Announces that the British Orthopaedic Association (BOA), Brighton Medical School and Sussex Medical School will send a questionnaire to BOA members to quantify perceptions and willingness to change surgical practice to reduce the carbon footprint. |
| **Dickson *et al.* 2022 [122]** | United Kingdom | Hand surgery (carbon neutral care) | Outlines five targeted and practical priorities for delivering carbon neutral hand surgery.  These priorities are; (i) recognising that hand surgery needs to be environmentally sustainable, (ii) measuring the current carbon footprint, (iii) performing research and innovation, (iv) establishing a paradigm shift in clinical practice and (v) ensuring that surgical waste is effectively reduced, reused or recycled. | Reports that hand surgery is well positioned to make changes to reduce carbon emissions related to practice, however, requires leadership and willingness to change. |
| **Evans 2021 [107]** | Australia | Podiatry (environmental impact) | Describes how podiatrists can target upstream factors in their clinic that produce greenhouse gas emissions by implementing changes such as telehealth, stocking sustainable footwear and educating patients on the importance of environmental sustainability. | Concludes that podiatrists can engage with their community to model positive environmental practices. As foot health consumers, podiatry patients are increasingly likely to expect more sustainable practices and products, including ‘green footwear’ options. |
| **Evans 2021 [108]** | Australia | Podiatry (sustainability) | Describes a sustainability panel session held during the 2021 Australian Podiatry Association conference. Guest speakers from the panel session discussed utilising waste as a resource for orthotics, eliminating care that has low-value – particularly care with a large carbon footprint, and recycling programs for shoes to promote environmental sustainability. | The 26th United Nations Climate Change Conference (COP26) in Glasgow 2021 is an important meeting for addressing the impacts of climate change on the health of humans and the planet. ‘Green podiatry’, where podiatrists engage in sustainable practices, is important to reduce the carbon footprint. |
| **Garcia Diaz & Richardson 2021 [112]** | Canada | Occupational therapy | Describes how occupational therapists can combat climate change and lifestyle diseases by framing climate change from a personal and public health perspective. | Suggests occupational therapists should tailor treatment plans with an environmentally sustainable focus in mind. Also encourages occupational therapists to advocate for improved physical environments and policies to encourage healthier choices within communities. |
| **Greer III 1994 [123]** | United States | Orthopaedic surgery (recycling) | Poses a question of whether physically undamaged screws or prostheses be cleaned and re-sterilized if it is determined they are not suitable for a patient after being opened or inserted during orthopaedic surgery. | Suggests a “standard of care” may have already been developed to address this question and if not, perhaps this should be done. |
| **Jones 2009 [110]** | Australia | Physiotherapy (environmental impact) | Suggests there is scope for a stronger approach to promoting environmentally sustainable physiotherapy practice. | Proposes that the physiotherapy profession should embrace a culture of environmental sustainability and should strive to become a carbon neutral profession. |
| **Kuvadia *et al.* 2020 [124]**  Records commenting on Kuvadia *et al.* [80, 81] | United States and Canada | Surgery (regional anaesthesia to reduce greenhouse gas emissions) | Contends that by avoiding volatile inhalational agent use in general anaesthesia, regional anaesthesia may reduce greenhouse gas emissions and help prevent global warming.  Provided a theoretical calculation to highlight that substituting regional anaesthesia for general anaesthesia in total hip and knee replacement surgeries for a single New York hospital would save an equivalent of 26,900 lbs of coal burned in one year (2019 data). | Concludes that substituting regional anaesthesia for general anaesthesia during knee and hip replacement surgery may reduce greenhouse gas emissions [124].  A letter to the editor [80] in response to this editorial suggested that some reported data were miscalculated or based on a misinterpretation. Updated data were provided.  Kuvadia *et al.* [81] responded to thank Özelsel *et al.* [80] for their updated calculations and reasoning for the changes. An explanation of earlier calculations was provided. |
| **Lee & Mears 2012 [126]**  Record commenting on Lee & Mears [82] | United States | Orthopaedic surgery (operating room) | Summarises the amount of waste produced in hospitals and operating rooms. Discusses waste disposal methods for reducing waste and recycling from orthopaedic surgery and how it can be improved. Describes the ‘Practice Greenhealth’ initiative that aims to reduce the operating room carbon footprint. | Concludes that orthopaedic surgeons need to be aware and actively involved in changes aimed at ‘greening the operating room’. Highlights that further research is required in this area and will be possible as more health care facilities adopt sustainable changes in practice. |
| **Lidgren 2020 [117]** | Sweden and United Kingdom | Orthopaedic surgery (operating room) | Estimates the financial benefits of recycling metal implants at revision and at post-mortem in Sweden and extrapolates to Europe and the US. | Sixty tonnes of metal implants have been recycled in Sweden from cremation since 2016, with a net value of US$15 million. It was estimated that US$250 million per year could be saved when applying these figures to Europe and the United States.  Suggests that metal implant manufacturers collaborate with orthopaedic societies to recycle implants. Proposes that some income from recycling be directed to musculoskeletal research. |
| **Maric & Nicholls 2019 [115]** | New Zealand | Physiotherapy (environmental impact) | Advocates that more consideration needs to be given to the way that physiotherapists can reduce some of the negative effects of health care on the environment. | Physiotherapy needs to explore the environmental impacts of physiotherapy practice and establish profession that considers the environmental impact of clinical practice by making decisions and creating processes that minimise environmental harm.  Suggests changes that may be needed to establish physiotherapy as an environmentally friendly profession. |
| **Maric *et al.* 2021 [109]** | Norway, United Kingdom, United States, New Zealand, Switzerland, Pakistan, Germany, Australia, Greece, Brazil, and Sweden | Physiotherapy (environmental impact) | Provides a report on the *Environmental Physiotherapy (EPT) Agenda 2023*, which was launched in March 2020.  The *EPT Agenda 2023* is a global call to action for education institutions to integrate planetary health, environmental and sustainability perspectives into entry-level physiotherapy programs. | Identifies that the *EPT Agenda 2023* is the largest international effort in physiotherapy to date, however, it does not include all relevant developments in the field.  Some physiotherapy programs not participating in the *EPT Agenda 2023* are driving change by actively integrating the SDGs into their programs using a bottom-up approach. |
| **Maric *et al.* 2021 [114]** | Norway and India | Physiotherapy (sustainability) | Describes how environmental sustainability relates to physiotherapy.  Proposes that the environmental footprint of physiotherapy interventions should be regularly measured as part of future research, and describes concepts related to measuring the environmental impact of physiotherapy including the ‘carbon footprint’.  Provides an example of how environmental reasoning may become relevant to clinical practice, where environmental footprints of different treatments or pathways of care are factored into decision making. | Measuring the environmental footprint of physiotherapy interventions is necessary to improve the sustainability of health care systems and contribute to society’s transition to a sustainable future. |
| **Maric *et al.* 2022 [116]** | Norway and USA | Physiotherapy | Outlines the connection between ecological health and human health. Describes how outdoor physical activity in environments with polluted air can unintentionally increase the incidence of non-communicable disease (e.g. carviovascular and respiratory) for future generations, and calls upon physiotherapists to “speak out” to address this issue. | Describes three action strategies that physiotherapists can implement to advance environmental stewardship; ‘connect’, ‘learn’ and ‘act’. The ‘act’ strategy encourages the promotion of healthy lifestyles and outdoor activity, the use of active modes of transport, as well as reducing the environmental footprint of health systems including physiotherapy. |
| **Palstam *et al.* 2021 [118]** | Sweden | Physiotherapy (environmental impact) | Argues that physical therapy can play an important role in reducing the human impact on the environment by providing treatment options that reduce the need for resources and pharmaceuticals, and by promoting behavioural changes that have positive effects on both health and the environment (e.g. physical activity). | Suggests outcomes of physical therapy are considered against a “triple bottom line” of environmental, social and financial costs. This editorial seeks to empower physical therapists globally to identify and evaluate measures of sustainability relevant to their specific field of research and contribute to the transition towards a more sustainable health sector. |
| **Shahi *et al.* 2021 [125]** | United States | Orthopaedic surgery (environmental impact) | Describes how direct and indirect greenhouse gas emissions associated with a product or service are reported in terms of a carbon footprint to quantify environmental impact. Provides an overview of the carbon footprint of orthopaedic surgery by reporting several previous studies included in this scoping review. | Concludes that the environmental impact of health care is a critical issue and needs to be addressed to minimise its carbon footprint.  Outlines that orthopaedic surgery is a significant contributor to greenhouse gas emissions, and further steps are required to measure and reduce its carbon footprint. |
| **Stanhope *et al.* 2021 [111]** | Australia and Norway | Physiotherapy (ecosystem services) | Introduces the concept of ‘ecosystem services’ and the role that physiotherapists can play to (i) improve the care of patients, (ii) improve population health by advocating for ecosystem conservation and ecological restoration and (iii) promote and enhance environmentally sustainable physiotherapy practice to reduce the environmental impact of care. | Encourages physiotherapists to consider the role of the environment on patient health and the impact that physiotherapy on the environment.  Concludes that physiotherapists have an opportunity to influence the health of their patients on an individual level, and also on a public health, ecosystem health and planetary sustainability level. |
| **Van Demark *et al.* 2018 [127]** | United States | Hand surgery (cost analysis and waste audit) | Provides a cost comparison of ‘Lean and Green’ hand surgery compared to standard surgery for Wide Awake Local Anaesthesia No Tourniquet (WALANT) anaesthesia.  The ‘Lean and Green’ approach to hand surgery includes; (i) local anaesthesia without tourniquet, (ii) ‘minor field sterility’, where less surgical materials such as draping, gowns and antibiotics are used in the operating theatre, and (iii) a customised surgical pack containing minimal surgical instruments.  Authors report that their review of 1,099 hand surgery cases performed between October 2014 and December 2016 reduced the weight of surgical waste by 2.8 tons when using customised surgical packs rather than standard packs. | Concludes that the ‘Lean and Green’ approach to hand surgery provides both financial and environmental benefits when compared to standard surgery, without compromising patient satisfaction or quality of care. |
