# Supplementary material for: The environmental impact of health care for musculoskeletal conditions: A scoping review
Source: PLoS One. 2022 Nov 28;17(11):e0276685. doi: 10.1371/journal.pone.0276685 (PMC9704655; doi:10.1371/journal.pone.0276685)
Supplement: S4 Table — (DOCX) [file pone.0276685.s006.docx]

**S4 Table. Characteristics and findings of literature reviews**

| **Author (year)** | **Country of author/s** | **Topic** | **Focus** | **Conclusions** |
| --- | --- | --- | --- | --- |
| **Bravo *et al.* 2020 [128]** | United States | Hand surgery (environmental impact) | Described and promoted the ‘Lean and Green’ initiative which was created by multiple American hand surgery associations and societies to reduce waste produced by hand surgery.  Summarises strategies to reduce the financial cost and environmental impact of hand surgery. This includes reducing energy consumption of surgical practice by installing more efficient equipment (e.g. lighting, heating and cooling), modifying sterilisation techniques to reduce waste, and recycling disposable materials. Other considerations were presented such as patient transport, type of anaesthesia used for hand surgery and sanitation. | Determining the magnitude and sources of waste and non-waste elements related to hand surgery are important to identify opportunities to reduce environmental impact.  This can be driven through educating hand surgeons, changing policies for practice within operating rooms, conserving energy and establishing incentives for wser reduction.  Carbon emissions of hand surgery can be quantified using tools such as an online calculator or open-source life cycle assessment software, which may be appropriate for hospitals and private practice. |
| **Engler *et al.* 2022 [129]** | United States | Orthopaedic surgery (environmental sustainability) | Discussed the role of healthcare in climate change and review the literature to present evidence related to environmental sustainability and orthopaedic surgery. | The effects of climate change have led to interest in mitigating and reducing greenhouse gas emissions across all healthcare sectors. There are several studies that have assessed the carbon footprint of orthopaedic surgery, although more high-level research is needed. |
| **Ma & Han 2022 [131]** | United Kingdom | Hand surgery (carbon neutral practice) | Introduced the “Reduce, Reuse, Recycle, Research, Rethink and Culture” framework to describe changes that can be implemented for sustainable hand surgery practice. | Small changes in hand surgery practice aimed at reducing environmental impact can lead to significant changes when implemented on a large scale.  Hand surgeons should move away from traditional operating room procedures and be environmentally responsible in clinical practice. |
| **Yadav *et al.* 2020 [130]** | India | Medical devices (orthopaedic implants) | Described the sustainability and circular economy of 3D printing medical implants using biomaterials for orthopaedic surgery, which can completely dissolve at a predefined rate of degradation (e.g. orthopaedic fracture fixation plates, nails and screws).  Discussed the biocompatibility and mechanical properties of different materials used to manufacture orthopaedic implants, including; metallic biomaterials, biodegradable polymers, bioinert and bioresorbable ceramics, alumina, zirconia, carbon, calcium hydroxyapatite, bio-glass and glass ceramics. | Concludes that bioresorbable implants are the solution for sustainability when compared to implants made from permanent biomaterials (metals, ceramics and polymers). A limitation exists where bioresorbable implants cannot be used on long bones where a high ultimate tensile strength is needed.  3D printable bioresorbable materials will establish an orthopaedic industry that is sustainable and oriented towards circular economy. |
