# Supplementary material for: The environmental impact of health care for musculoskeletal conditions: A scoping review
Source: PLoS One. 2022 Nov 28;17(11):e0276685. doi: 10.1371/journal.pone.0276685 (PMC9704655; doi:10.1371/journal.pone.0276685)
Supplement: Attachment — Submitted filename: Response to Reviewers.docx [file pone.0276685.s007.docx]

Dear Professor Meyer,

Thank you for your further feedback of our paper titled: The environmental impact of health care for musculoskeletal conditions: a scoping review, PONE-D-22-02641. We have uploaded a revised manuscript with and without track changes that includes all of your suggestions relating to the results, discussion and conclusion sections. Your feedback was very helpful and we believe has improved our review.

Point-by-point responses to your feedback are outlined below.

**TABLE 1**

***Wang et al.
Total surgeries: 100 by a single surgeon (50 s general and 50 spinal anaesthesia)
“S” after 50 is a typo***

**Authors' response:** Thank you for picking up this typo. It has been corrected in the manuscript.

***Median total carbon footprint, grams CO2e Spinal anaesthesia12: 70
70g of CO2 seems to be too little--is this just considering the spinal needle and the local anesthetic? What about the drapes, O2 nasal cannula, O2 gas…? Especially when compared to the McGain Anesthesiology paper (and these two should be discussed together), there is a contradiction in findings.

Similarly 231-234 …one retrospective study from the United States reported that the median total carbon footprint of general anaesthesia was significantly higher when compared to spinal anaesthesia (4,725 grams CO2e versus 70 grams CO2e) for a single-level transforaminal lumbar interbody fusion [53].***

**Authors' response:** Thank you for highlighting the apparent inconsistencies of results between the McGain et al. (2021) and Wang et al. (2022) studies.

We agree that the 70 grams of CO_2_e emissions for spinal anaesthesia during single-level TLIFs as reported by Wang et al. is small but is likely explained by the study design which was a partial LCA known as a “cradle-to-gate” assessment. This method includes the carbon footprint of a product from the resource extraction phase (“cradle”) to the moment it is sold or received by the consumer (“gate”). Therefore, the environmental impact of anaesthetic machines, cleaning equipment, compression of liquid oxygen, plastic disposable items or waste disposal was not considered.

In contrast, the higher values reported by McGain et al. is because their LCA was a “cradle-to-grave” assessment. This includes the carbon footprint associated with using the product and its disposal (“grave”).

Another reason for the discrepancy in CO_2_e emissions is likely explained by different sources of electricity used in Australia and the United States. According to McGain et al., electricity generated in Victoria, Australia generates twice the CO_2_e emissions when compared to the US).

We have updated the results section to include an explanation of the likely reasons for the differing results (please see lines 304 to 313).

**RESULTS**

***In all results subsections, but specifically “original research studies” please collate and present consistent themes from the conclusions of the manuscripts. The results section reads like a bibliography (specifically sentence two of paragraph one (214-226) and presents the topic of the article found, but very little about the lessons of that article. You have done the identification of the important points in “results” and “conclusions” columns in Table 1. Please put these conclusions together for the reader in results to assist in hypothesis-generation for future research.***

***As an example, additive manufacturing is mentioned at least twice as being less carbon intensive. This is a new-ish technology which has multiple applications in the medical world--I was not aware how much more sustainable it is considered--there is value in sharing this.***

**Authors' response:** We had not wanted to overburden the reader by duplicating the results and conclusions columns of Table 1 in the text. However, we agree that reporting more details from the original studies is worthwhile and we have now included this information in the text of the manuscript for all LCA and carbon footprint studies methods (see lines 225 to 313). We have also updated the results for the two LCAs that reported a significantly reduced environmental impact of additive manufacturing compared to conventional methods (see lines 225 to 231).

We did not expand the editorial and narrative review subsections of the results as their details are described in Tables S3 and S4 but we can do so if the editor wishes us to.

**DISCUSSION**

***333-344 are two very complicated sentences requiring simplification for comprehension.***

**Authors' response:** We agree and have now revised and simplified these sentences (see lines 451 to 465).

**CONCLUSION**

***The following sentence isn't quite right because it implies we should expect clarity in other segments of health care whereas the assessments and connections are frustratingly basic in health care and other industries--linking GHG emissions to any complex process is very challenging.

“Despite an established link between health care and greenhouse gas emissions, the carbon footprint of health care for musculoskeletal conditions is unknown.”***

**Authors' response:** We agree that this is a complex process. We have now revised the conclusion to better match the conclusion in the abstract (see lines 502 to 509). We have also added text to the third paragraph of the discussion indicating that measuring the environmental impact of health care is complex and requires specialised expertise (see lines 428 to 431).

**You have now found some manuscripts with the additional research--what did you find, and what do you want to be the specific next steps?**

We have added a paragraph in the discussion to summarise what we found overall and promising avenues for further research (see lines 406 to 416).
